# Skeletal muscle releases extracellular vesicles with distinct protein and microRNA signatures that function in the muscle microenvironment

**DOI:** 10.1093/pnasnexus/pgac173

**Published:** 2022-08-26

**Authors:** Sho Watanabe, Yuri Sudo, Takumi Makino, Satoshi Kimura, Kenji Tomita, Makoto Noguchi, Hidetoshi Sakurai, Makoto Shimizu, Yu Takahashi, Ryuichiro Sato, Yoshio Yamauchi

**Affiliations:** Laboratory of Food Biochemistry, Department of Applied Biological Chemistry, Graduate School of Agricultural and Life Sciences, The University of Tokyo, Tokyo 113-8657, Japan; Laboratory of Food Biochemistry, Department of Applied Biological Chemistry, Graduate School of Agricultural and Life Sciences, The University of Tokyo, Tokyo 113-8657, Japan; Laboratory of Food Biochemistry, Department of Applied Biological Chemistry, Graduate School of Agricultural and Life Sciences, The University of Tokyo, Tokyo 113-8657, Japan; Technology Advancement Center, Graduate School of Agricultural and Life Sciences, The University of Tokyo, Tokyo 113-8657, Japan; Technology Advancement Center, Graduate School of Agricultural and Life Sciences, The University of Tokyo, Tokyo 113-8657, Japan; Nutri-Life Science Laboratory, Department of Applied Biological Chemistry, Graduate School of Agricultural and Life Sciences, The University of Tokyo, Tokyo 113-8657, Japan; Center for iPS Cell Research and Application, Kyoto University, Kyoto 606-8507, Japan; Nutri-Life Science Laboratory, Department of Applied Biological Chemistry, Graduate School of Agricultural and Life Sciences, The University of Tokyo, Tokyo 113-8657, Japan; Laboratory of Food Biochemistry, Department of Applied Biological Chemistry, Graduate School of Agricultural and Life Sciences, The University of Tokyo, Tokyo 113-8657, Japan; Laboratory of Food Biochemistry, Department of Applied Biological Chemistry, Graduate School of Agricultural and Life Sciences, The University of Tokyo, Tokyo 113-8657, Japan; Nutri-Life Science Laboratory, Department of Applied Biological Chemistry, Graduate School of Agricultural and Life Sciences, The University of Tokyo, Tokyo 113-8657, Japan; AMED-CREST, Japan Agency for Medical Research and Development, Tokyo 100-0004, Japan; Laboratory of Food Biochemistry, Department of Applied Biological Chemistry, Graduate School of Agricultural and Life Sciences, The University of Tokyo, Tokyo 113-8657, Japan; Nutri-Life Science Laboratory, Department of Applied Biological Chemistry, Graduate School of Agricultural and Life Sciences, The University of Tokyo, Tokyo 113-8657, Japan; AMED-CREST, Japan Agency for Medical Research and Development, Tokyo 100-0004, Japan

**Keywords:** extracellular vesicles, exosomes, skeletal muscle, interstitium

## Abstract

Extracellular vesicles (EVs) contain various regulatory molecules and mediate intercellular communications. Although EVs are secreted from various cell types, including skeletal muscle cells, and are present in the blood, their identity is poorly characterized *in vivo*, limiting the identification of their origin in the blood. Since skeletal muscle is the largest organ in the body, it could substantially contribute to circulating EVs as their source. However, due to the lack of defined markers that distinguish skeletal muscle-derived EVs (SkM-EVs) from others, whether skeletal muscle releases EVs *in vivo* and how much SkM-EVs account for plasma EVs remain poorly understood. In this work, we perform quantitative proteomic analyses on EVs released from C2C12 cells and human iPS cell-derived myocytes and identify potential marker proteins that mark SkM-EVs. These markers we identified apply to *in vivo* tracking of SkM-EVs. The results show that skeletal muscle makes only a subtle contribution to plasma EVs as their source in both control and exercise conditions in mice. On the other hand, we demonstrate that SkM-EVs are concentrated in the skeletal muscle interstitium. Furthermore, we show that interstitium EVs are highly enriched with the muscle-specific miRNAs and repress the expression of the paired box transcription factor *Pax7*, a master regulator for myogenesis. Taken together, our findings confirm previous studies showing that skeletal muscle cells release exosome-like EVs with specific protein and miRNA profiles *in vivo* and suggest that SkM-EVs mainly play a role within the muscle microenvironment where they accumulate.

Significance Statement Various cells/tissues release extracellular vesicles (EVs) that encapsulate diverse regulatory molecules, including miRNAs and proteins, thereby mediating cell–cell communications. EVs are present abundantly in the blood through which they are transported between tissues. However, little is known about *in vivo* dynamics of certain EVs because tissue-specific EV markers have been poorly characterized. Here, we confirm the distinct protein and miRNA signatures of skeletal muscle-derived EVs (SkM-EVs). By tracking SkM-EV markers, we demonstrate that SkM-EVs accumulate within the muscle microenvironment, rather than enter the circulation. We further show that SkM-EVs regulate myogenic gene expression in myoblasts. Our results demonstrate the paracrine action of SkM-EVs within skeletal muscle tissue, providing a conceptually new basis for how SkM-EVs exert their functions.

## Introduction

Skeletal muscle is the largest organ in the body, accounting for 40% of body weight and is responsible for locomotion activity, whole-body metabolism, and energy homeostasis. Moreover, skeletal muscle serves as a secretory organ (1, 2); it secretes various humoral factors known as myokines, including irisin, apelin, interleukins, and myostatin. They act as mediators for cell–cell communications in autocrine, paracrine, and endocrine fashions. Each myokine has distinct functions and influences tissue homeostasis and metabolism within skeletal muscle and in other tissues (1, 2). Exercise can induce the expression and secretion of some myokines, which partly explains the health benefits of exercise (2, 3). Thus, skeletal muscle is considered as an important secretory organ that governs whole-body homeostasis.

In addition to humoral factors, cells release membrane vesicles to the extracellular milieu. Over the last decade, much attention has been paid to the extracellular vesicles (EVs) because they accommodate a wide variety of bioactive molecules, including nucleic acids [DNA, mRNA, microRNA (miRNA), and long noncoding RNA], proteins, lipids, and metabolites, and deliver them to recipient cells (4–8). Thus, EVs also act as a means for intercellular and interorgan communications in physiological and pathophysiological settings, including exercise, cancer, and metabolic diseases (9, 10). EVs are heterogeneous in nature and classified into three classes based on size and biogenesis mechanisms, exosomes (50 to 150 nm in diameter), microvesicles (100 to 500 nm), and apoptotic bodies (100 to 5,000 nm) (4, 7, 8). Exosomes are derived from the multivesicular bodies (MVBs) of the late endosome. The MVBs fuse with the plasma membrane (PM) and intraluminal vesicles (ILVs) inside the MVBs are released to the extracellular environment as exosomes. Microvesicles are originated from the plasma membrane by membrane budding. Apoptotic bodies are released from apoptotic cells. EVs are abundantly present in body fluids, including plasma. It is thus expected that their constituents serve as useful biomarkers for diagnosis (10, 11). On the other hand, once they are released from original tissues and enter the circulation, it is nearly impossible to identify their origin because tissue-specific EV markers are poorly characterized. This issue makes it difficult to understand the contribution of each tissue to circulating EVs and to track certain EVs *in vivo*.

Like other cell types, skeletal muscle cells are capable of releasing EVs (12). Evidence shows that C2C12 murine myoblasts and myotubes, and human primary myocytes release EVs (13–15). EVs released from C2C12 myotubes are transferred to myoblasts and regulate differentiation into myotubes by modulating gene expression (15, 16). Furthermore, EVs derived from C2C12 myotubes contain miRNAs specifically or abundantly expressed in skeletal muscle called myomiRs (16, 17) that regulate skeletal muscle homeostasis (18, 19). These data suggest that miRNAs from SkM-EVs have physiological functions. Although previous papers identified specific proteins as markers of SkM-EVs (ITGA7 (13) and SPARC (20)), the potential of SkM-EVs proteins to follow their trafficking *in vivo* has been poorly explored. Due to the lack of defined SkM-EV markers, whether skeletal muscle actively releases EVs *in vivo*, how much proportion of plasma EVs are derived from this tissue, and where SkM-EVs are delivered and exert their roles remain largely unknown. In addition, although recent studies show that exercise increases circulating EVs (21–23) and that SkM-EVs are released into the blood (24), it is under debate whether skeletal muscle contributes to the exercise-dependent increase in circulating EVs.

To address these issues, here we seek to identify SkM-EV marker proteins by quantitative proteomics on human and mouse myocyte-derived EVs and investigate whether skeletal muscle releases exosome-like small EVs *in vivo*. Based on our proteomic profiling of EVs released from these myocytes, we provide *in vivo* evidence that skeletal muscle actively releases small EVs with distinct protein and miRNA profiles and that SkM-EVs highly accumulate within the skeletal muscle interstitium rather than being secreted into the blood. We further show that EVs isolated from the muscle interstitium modulate myogenic gene expression in murine myoblasts. We thus propose that SkM-EVs mainly exert their functions within the muscle microenvironment.

## Results

### C2C12 cells and hiPSC-derived myocytes secrete EVs

**Fig. 1.**
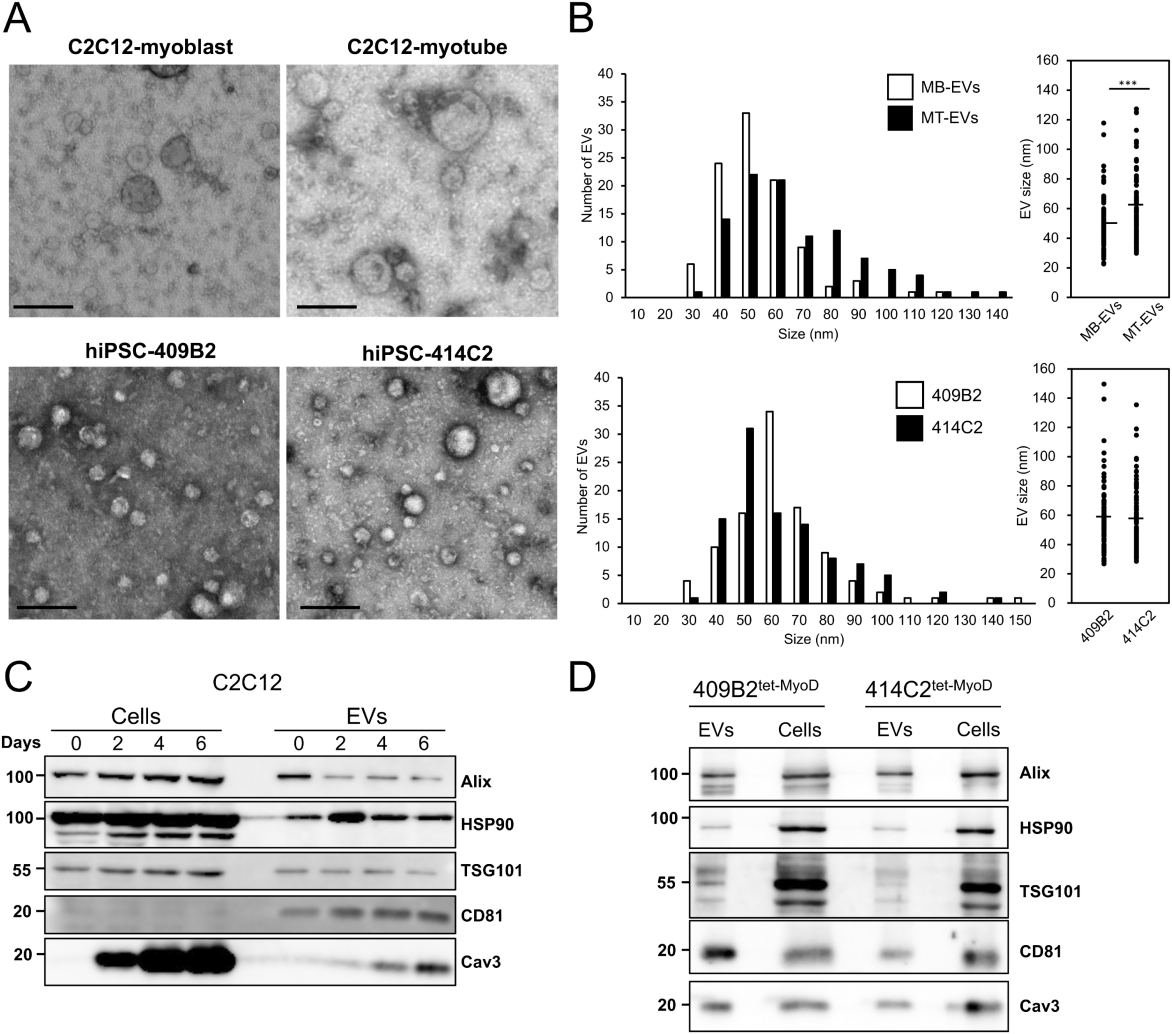
Isolation and characterization of EVs from cultured human and mouse myocytes. (A) TEM images of EVs. EVs were isolated by ultracentrifugation from C2C12 myoblasts, C2C12 myotubes, and two lines of hiPSC-myocytes (409B2^tet-MyoD^ and 414C2^tet-MyoD^) and images were acquired under TEM. Scale bar, 100 nm. (B) Size distribution of EVs. Sizes of EVs from C2C12-MB-EVs, C2C12-MT-EVs (top), and hiPS-MC-EVs (bottom) were measured using TEM images. Statistical analysis was performed by Student's *t*-test. ***, *P* < 0.005 (*n* = 100). (C) Differentiation-dependent expression of proteins in C2C12 cells and C2C12-derived EVs. Cell lysates and EVs were prepared on days 0, 2, 4, and 6. Forty-eight hours before harvest, medium was switched to EV-free medium. EVs were isolated from conditioned medium by ultracentrifugation as described in the “Materials and Methods” section. Expression of EV marker proteins in cell lysate (20 μg protein) and EVs (1 μg protein) was analyzed by immunoblot. (D) Protein expression in hiPSC-myocytes and their EVs. hiPSCs (409B2^tet-MyoD^ and 414C2^tet-MyoD^) were differentiated into myocytes. Afterward, myocytes were incubated for 48 h in medium containing 5% EV-free HS. EVs were isolated from conditioned medium by ultracentrifugation. Cell lysate (5 μg protein) and EVs (1 μg protein) were subjected to immunoblotting to analyze the expression of the indicated proteins.

To characterize EVs secreted from both human and mouse skeletal muscle cells, we first isolated EVs from mouse C2C12 myoblasts and myotubes, and human induced pluripotent stem cell (hiPSC)-derived myocytes (hiPSC-myocytes) by a standard ultracentrifugation protocol (25). C2C12 myoblasts were differentiated into myotubes (Fig. S1A and B) and incubated for 48 h in a differentiation medium containing EV-free horse serum (HS) before isolating EVs from the conditioned medium. To isolate C2C12 myoblast EVs, the cells were incubated for 48 h in an EV-free FBS medium. In addition, we used two lines of hiPSC-myocytes, 414C2^tet-MyoD^ and 409B2^tet-MyoD^. These hiPSC lines harbor tetracycline-inducible human *MYOD1* expressing *piggyBac* vector, and thus adding doxycycline (Dox) into culture medium induces MyoD1 expression, initiating myogenic differentiation. After 5 to 6 days after Dox addition, these hiPSCs differentiated into myocytes (Fig. S1C) expressing skeletal muscle cell marker proteins, including myosin heavy chain (MyHC), myogenin, and caveolin-3 but no longer expressing the iPSC marker proteins Nanog, OCT-4A, and Sox 2 (Fig. S1D). After differentiation, hiPSC-myocytes were incubated in a medium supplemented with EV-free HS for 48 h to isolate EVs from a conditioned medium. To observe the morphology of isolated EVs, we first performed transmission electron microscopy (TEM) analysis on EVs from C2C12 myoblasts (C2C12-MB-EVs), C2C12 myotubes (C2C12-MT-EVs), and hiPSC-myocytes (hiPS-MC-EVs). Figure 1A shows typical images of C2C12-MB-EVs, C2C12-MT-EVs, and hiPS-MC-EVs. The diameters of C2C12-MB-EVs and C2C12-MT-EVs were 48.8 ± 16.4 nm and 61.4 ± 22.4 nm, respectively (Fig. 1B). C2C12-MT-EVs were statistically larger than C2C12-MB-EVs. The average size of hiPS-MC-EVs was approximately 58 nm in both 414C2^tet-MyoD^ and 409B2^tet-MyoD^ lines (Fig. 1B). The sizes are all within the range of typical exosomes. Together, these results showed that both human and mouse skeletal muscle cells release small EVs with similar size.

We next examined the presence of the exosome marker proteins in the isolated EVs. The results show that C2C12-MB-EVs and C2C12-MT-EVs contained the well-defined exosome markers, including Alix, TSG101, CD81, and HSP90 (Fig. 1C). hiPS-MC-EVs also contained these exosome markers (Fig. 1D). In addition to these typical markers, we found that C2C12-MT-EVs and hiPS-MC-EVs but not C2C12-MB-EVs, contained caveolin-3, a protein highly expressed in skeletal muscle and cardiomyocytes, and its contents increased by differentiation. The results suggest that skeletal muscle cells release EVs harboring skeletal muscle-specific proteins.

### Proteomic profiling of EVs released from skeletal muscle cells

**Fig. 2.**
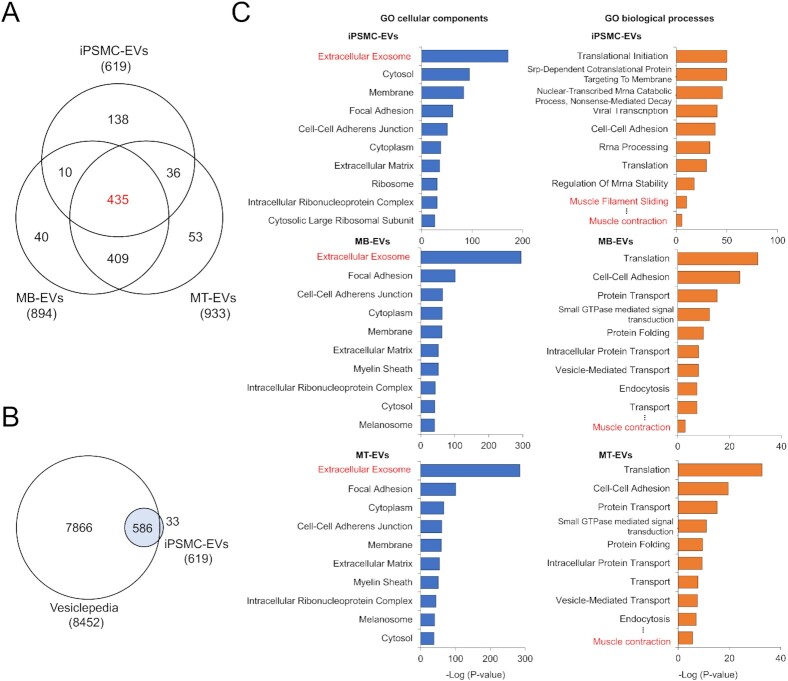
Proteomic profiling of myocyte-derived EVs. (A) Venn diagram showing the distinct and overlapping EV proteins from C2C12 myoblasts, C2C12 myotubes, and hiPSC-myocytes. Proteomic analyses were performed on EVs isolated from C2C12 myoblasts (MB-EVs), C2C12 myotubes (MT-EVs), and hiPSC-myocytes (iPSMC-EVs) by ultracentrifugation. The 619 proteins in iPS-MC-EVs were consistently detected in EVs secreted from 414C2^tet-Myo-D^ and 409B2^tet-MyoD^. (B) Venn diagram showing proteomic coverage of hiPS-MC-EVs versus Vesiclopedia database. (C) GO analysis of myocyte-derived EVs for cellular components (left) and biological processes (right). Proteomic data on iPS-MC-EVs (top), C2C12-MB-EVs (middle), and C2C12-MT-EVs (bottom) were analyzed using DAVID. Top 10 GO term are listed.

To determine proteomic profiling of EVs released by skeletal muscle cells, we first performed quantitative shotgun proteomic analyses on C2C12-MB-EVs and C2C12-MT-EVs isolated by ultracentrifugation (Table S1). The analyses identified 894 and 933 proteins in C2C12-MB-EVs and C2C12 MT-EVs, respectively, which cover 983 different proteins (Fig. 2A). Previously, Forterre et al. identified 455 proteins as those found in EVs secreted from C2C12 myoblasts and myotubes (15). Of the 455 proteins, 354 proteins (78%) were also found in our results (Fig. S2A). The current results thus revealed 629 additional C2C12-MB/MT-EVs proteins not identified previously.

Next, the same proteomic analysis was performed on hiPS-MC-EVs from both 409B2^tet-MyoD^ and 414C2^tet-Myo-D^ lines (Table S2), and 624 and 628 proteins were detected in those from 409B2^tet-MyoD^ and 414C2^tet-Myo-D^, respectively, showing over 98.5% of EV proteins overlaps (Fig. S2B). Among the 619 proteins identified in hiPS-MC-EVs, the 586 proteins (95%) are covered by the EV database Vesiclepedia (26) (http://microvesicles.org), validating isolated EVs of quality (Fig. 2B). Of the 619 proteins, 78% (481 proteins) were also found in EVs isolated from either C2C12-MB-EVs or C2C12-MT-EVs (Fig. 2A). The results also identified 435 proteins that overlap among C2C12-MB-EVs, C2C12-MT-EVs, and hiPS-MC-EVs. Thirty-six proteins were found in both C2C12-MT-EVs and hiPSM-EVs but not in C2C12-MB-EVs (Table S3), suggesting a distinct protein profile of myotube-derived EVs. On the other hand, 40 proteins were found only in C2C12-MB-EVs (Table S4). We next compared our results with published results which reported the identification of 954 proteins secreted from differentiating human myoblast through either classical secretory pathway or unconventional mechanisms that include EVs (14). Of the 954 proteins, 238 proteins (25%), and 340 proteins (36%) were found in our hiPS-MC-EVs (Fig. S2C) and C2C12-MB/MT-EVs (Fig. S2D), respectively. Our proteomics data along with others suggest that human and mouse SkM-EVs exhibit similar protein profiles.

To annotate identified EV proteins, we classified these proteins based on Gene Ontology (GO) using an integrative platform, DAVID (27, 28). The results showed that in hiPS-MC-EVs, C2C12-MB-EVs, and C2C12-MT-EVs, proteins belonging to the term “Extracellular Exosome” in “Cellular Components” were highly enriched, confirming that isolated EVs are of good quality (Fig. 2C). Consistent with previous studies (14), among the established EV/exosome markers tetraspanins, CD9, CD63, and CD81 (4, 7, 10), our proteomics identified only CD81 in both C2C12-MB/MT-EVs (Table S1) and hiPS-MC-EVs (Table S2). For the “Biological processes” term, proteins classified into “Muscle contraction” were significantly enriched in all three EV samples, which indicates that SkM-EVs contain proteins unique to skeletal muscle. Together, all these results suggest that SkM-EVs display a distinct protein signature.

### Identification of potential marker proteins for SkM-EVs

**Fig. 3.**
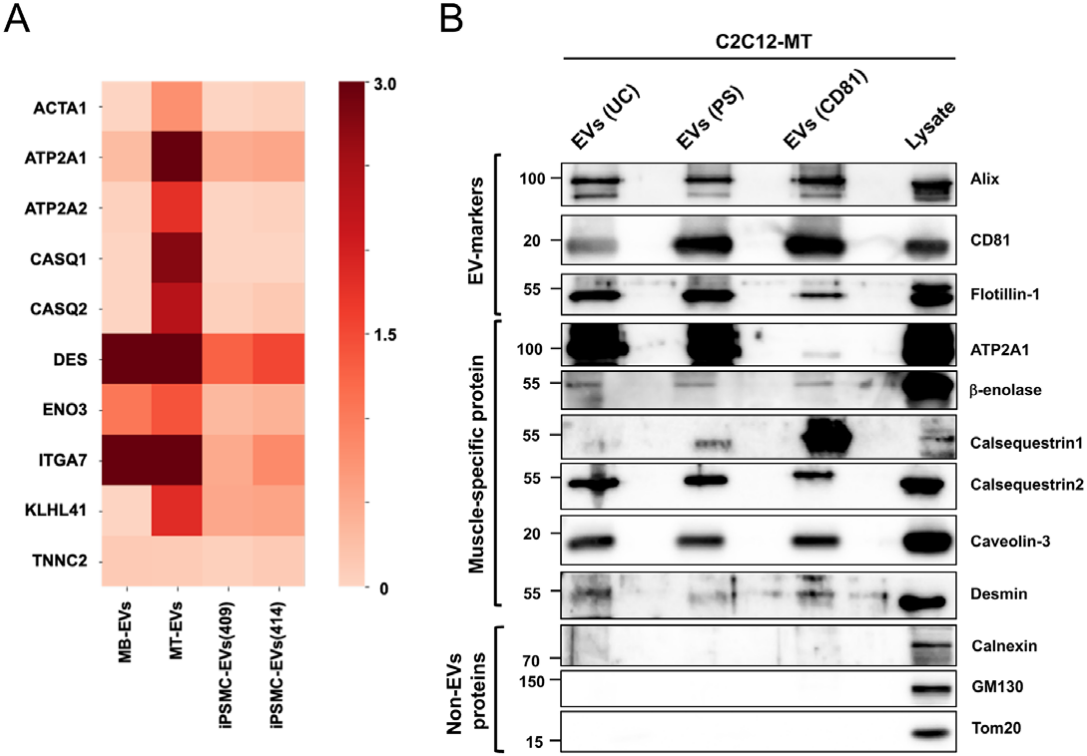
Identification of potential SkM-EV markers *in vitro*. (A) Heatmap showing the contents of potential SkM-EV markers in EVs isolated by ultracentrifugation. The contents of the ten muscle-specific proteins in C2C12-MB-EVs (MB-EVs), and C2C12-MT-EVs (MT-EVs), and hiPS-MC-EVs (iPSMC409-EVs and iPSMC414-EVs) are shown. Skeletal muscle proteins were selected based on the public databases, Human Protein Atlas and GTEx. (B) Expression of EV-marker proteins in C2C12-MT-EVs. C2C12-MT-EVs were isolated by ultracentrifugation (UC), PS-affinity beads (PS), and CD81-affinity beads (CD81). The expressions of typical EV marker proteins and muscle-specific proteins in EV fractions and cell lysate were analyzed by immunoblot.

We next sought to identify potential marker proteins that mark EVs released from skeletal muscle cells. To this end, we searched proteins highly expressed in skeletal muscle from our proteome data obtained from hiPS-MC-EVs and C2C12-MT-EVs. As mentioned above, we identified 36 potential MT-EV proteins (Fig.   2A, Table S3). To assess their specificity, we searched specific proteins using the Gene Ontology Consortium's Community Annotation Wiki for Muscle Biology (http://wiki.geneontology.org/index.php/Muscle_Biology) and confirmed that many of these proteins, including Nebulin, KLHL41, MYH1, TRIM72, ACTA1, and MYBPH are predominantly expressed in skeletal muscle. Furthermore, based on The Human Protein Atlas and The Genotype-Tissue Expression (GTEx) databases, we selected 10 proteins as potential marker proteins for SkM-EVs (Fig.   3A). To confirm whether these proteins are included in EVs, C2C12-MT-EVs were isolated by three methods, standard ultracentrifugation (UC-EVs), the Tim4-based method, which is based on the high-affinity binding of Tim4 to phosphatidylserine (PS) (PS^+^ EVs) (29), and immuno-isolation with anti-CD81 antibody (CD81^+^ EVs), and subjected to immunoblot analysis. Due to the availability and/or validity of antibodies, six out of ten proteins were analyzed. The results show that in addition to the typical exosome marker proteins (Alix, CD81, and Flotillin-1), C2C12-MT-EVs contain the skeletal muscle proteins, ATP2A1, β-enolase, calsequestrin 2, caveolin-3, and desmin (Fig. 3B), validating our proteomic analysis. None of the EVs isolated by the three methods contained detectable levels of calnexin (an ER marker), GM130 (a Golgi marker), and Tom20 (a mitochondrial marker). Intriguingly, CD81^+^ EVs contained much less ATP2A1 compared to UC-EVs and PS^+^ EVs but were highly enriched with calsequestrin 1, suggesting that CD81^+^ EVs show a distinct protein profile. Among the skeletal muscle proteins, ATP2A1, β-enolase, and desmin are predominantly expressed in skeletal muscle tissues (Fig. S3).

### SkM-EVs accumulate in the skeletal muscle interstitium

**Fig. 4.**
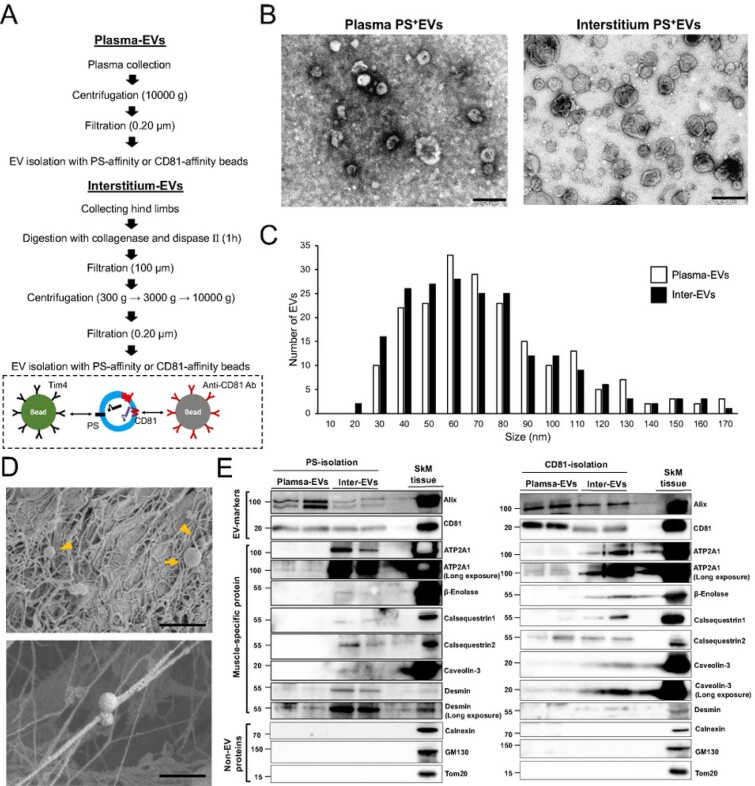
Validation of SkM-EVs markers *in vivo*. (A) Outlines of EV isolation protocols from plasma and the skeletal muscle interstitium. See the “Material and Methods” section for more detail. (B) TEM images of plasma and interstitium EVs. EVs were isolated using PS-affinity beads. Scale bar, 200 nm. (C) Size distribution of plasma and interstitium PS^+^ EVs. Size of EVs were analyzed using Image J (*n* = 200). (D) SEM images of skeletal muscle tissue (gastrocnemius). Small and large EVs are indicated arrowheads and arrows, respectively. The bottom image shows small EVs attaching ECM-like structures. Scale bar, 1 μm in upper panel and 500 nm in lower panel. (E) Expression of the EV marker proteins in plasma and SkM-interstitium EVs. Plasma and interstitium EVs were isolated from two mice using PS-affinity (left) or CD81-affinity (right) beads as in (A). Skeletal muscle tissue (quadriceps) homogenates were also prepared from the same mice. Plasma EVs (5 μg protein/lane) and interstitium EVs (Inter-EVs) (5 μg protein/lane) were subjected to immunoblot analysis to validate the presence or absence of the marker proteins in these EVs. SkM tissue homogenates (2 μg protein/lane) were also analyzed as positive controls.

Recent reports showed that apart from plasma, the interstitium of tissues such as the liver and lung contain significant amounts of EVs (30, 31). To determine whether the skeletal muscle cells release EVs *in vivo*, we isolated EVs from both plasma and skeletal muscle (tibialis anterior, gastrocnemius, soleus, and quadriceps) interstitium of mice using the Tim4-based method (Fig. 4A). We validated the quality of isolated EVs by TEM and confirmed that PS^+^ EVs from both the plasma and SkM-interstitium show similar morphology (Fig. 4B). Plasma and SkM-interstitium PS^+^ EVs were similar in size, ranging from 30 to 150 nm (Fig. 4C). Scanning electron microscopy (SEM) analysis showed that the skeletal muscle interstitium contains EV-like vesicles with a diameter of 50 to 500 nm, which are attached to extracellular matrix (ECM)-like structures (Fig. 4D). We next asked whether plasma and SkM-interstitium EVs contain SkM-EV markers identified above. We isolated EVs from plasma and SkM-interstitium using PS-affinity or CD81-affinity beads used to isolate C2C12-MT-EVs (Fig. 4A). To validate and compare the two methods, we first examined whether plasma and interstitial EVs isolated by these methods were contaminated with non-EV membranous structures. The results showed that non-EV proteins (calnexin, GM130, and Tom20) were undetectable in isolated EVs (Fig. 4E). We also examined the presence of lipoprotein markers in these fractions. The results showed that apolipoprotein A-I (apoA-I), a major apolipoprotein of high-density lipoprotein, was detected in plasma CD81^+^ EVs but not in the interstitial CD81^+^ EVs (Fig. S4A). ApoA-I was also found in plasma and interstitial PS^+^ EVs (Fig. S4B). ApoE was hardly detected in both plasma and interstitial EVs isolated by either method. Although whether apoA-I found in EV fractions are contamination or an EV component cannot be determined at present (32–35), we conclude that both methods can efficiently isolate EVs from plasma and interstitium. As expected, the typical EV markers Alix and CD81 were found in both plasma and SkM-interstitium EVs regardless of the isolation methods (Fig. 4E). In addition, potential SkM-EV marker proteins (ATP2A1, β-enolase, calsequestrin 1, calsequestrin 2, caveolin-3, and desmin) were detected at a different degree in PS^+^ and CD81^+^ EVs isolated from SkM-interstitium. In contrast, the SkM-EV markers were much less or undetectable in the plasma EVs. ATP2A1, calsequestrin 2, and desmin were slightly detected in plasma EVs, suggesting that SkM-EVs only partly enter the bloodstream. Calsequestrin 2-positive EVs found in the plasma could be derived from the heart where its expression is much higher than in skeletal muscle (Fig. S3). Altogether, our results indicate that ATP2A1, β-enolase, and desmin may serve as reliable SkM-EV protein markers *in vivo* and that SkM-EVs are highly concentrated in skeletal muscle tissues but are not major populations in the circulation.

**Fig. 5.**
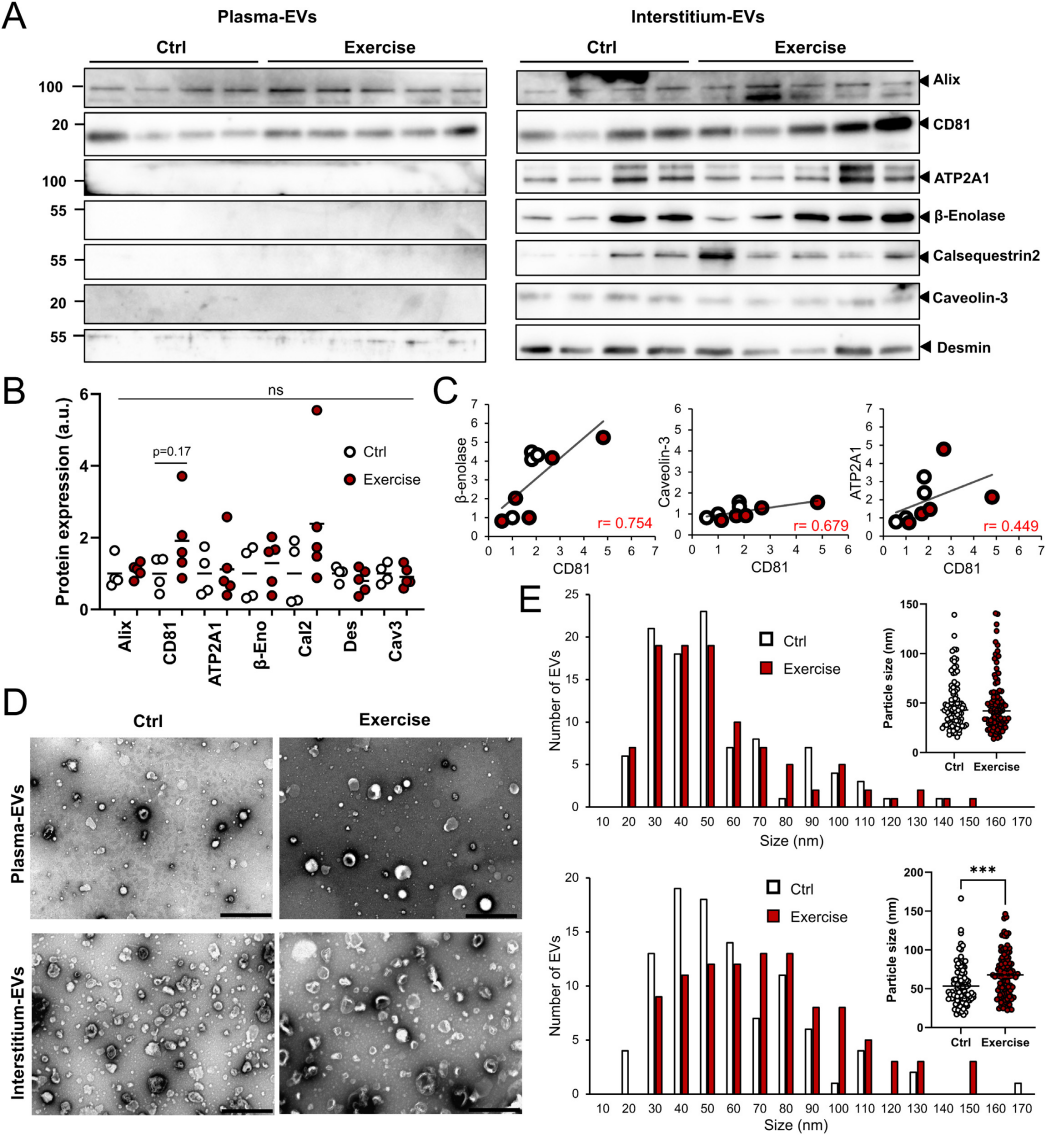
Effect of exercise on plasma and SkM interstitium EVs. (A) Immunoblot analysis of EVs. Plasma and skeletal muscle (hind limbs) interstitium EVs were isolated using PS-affinity beads from control (*n* = 4) and exercised mice (*n* = 5). Equal volume of plasma EVs (20 μL/lane, equivalent to EVs from 60 μL plasma) and interstitium EVs (20 μL/lane, equivalent to EVs from 60 mg tissue) were subjected to immunoblotting to detect the indicated proteins. Expression of these proteins in skeletal muscle tissues with or without exercise are shown in Figure S4C. (B) Quantification of protein levels in interstitium EVs before and after exercise. (C) Correlation between CD81 and SkM-EV marker proteins (β-enolase, caveolin-3, and ATP2A1). Each circle represents individual mice with (red circle) or without (white circle) exercise. (D) TEM images of plasma and interstitium EVs with or without exercise. Scale bar, 500 nm. (E) Size distribution of plasma (upper) and interstitium (lower) EVs isolated from mice with or without exercise. Statistical analysis was performed by Student's *t*-test. ***, *P* < 0.005 (*n* = 100).

To further determine the physiological importance of SkM-EVs *in vivo*, we examined the effect of exercise on SkM-EVs. Whether exercise increases circulating EV contents is currently controversial (21–23, 36–38). Moreover, even though exercise increases circulating EVs, their origin(s) is not fully characterized. We took advantage of our newly identified SkM-EV marker proteins to clarify this issue. After mice were subjected to exhaustive endurance running on a treadmill (Fig. S5A and B), we immediately harvested blood from the heart and skeletal muscle tissues from a hind limb, and prepared plasma EVs and SkM-interstitium EVs, respectively, using PS-affinity beads. The results showed that the single bout of acute exercise does not alter the concentration of proteins in either plasma EVs (Ctrl, 340.3 ± 22.5 mg/mL; Exercise, 328.4 ± 8.0 mg/mL; *P* = 0.30) or SkM-interstitium EVs (Ctrl, 371.4 ± 19.5 mg/mL; Exercise, 378.7 ± 23.6 mg/mL; *P* = 0.35). We also assessed levels of marker proteins in plasma and the interstitium EVs. Neither typical EV markers nor SkM-EV markers in plasma and interstitium EVs were significantly changed by the exercise, while trends in the increase by the exercise of the typical EV markers CD81 (1.4-fold; *P* = 0.25) and Alix (1.4-fold; *P* = 0.22) in plasma EVs and CD81 (2.0-fold; *P* = 0.17) in interstitium EVs were observed (Fig. 5A and B). CD81 and Alix expression decreased in the muscle after the exercise through an unknown mechanism (Fig. S5C). On the other hand, the exercise did not influence the expression of SkM-EV marker proteins in skeletal muscle (Fig. S5C). These results suggest that the acute exercise does not influence EV release from skeletal muscle. Meanwhile, we observed positive correlations between CD81 and β-enolase, caveolin-3, or ATP2A1 contained in the interstitium EVs (Fig.   5C, Table S5). It could be consistent with previous studies that skeletal muscle cells preferentially release CD81-positive EVs (12, 14). Furthermore, we noticed subtle but significant increases in the size of the interstitium EVs but not of plasma EVs upon exercise (Fig. 5D and E). Collectively, our results show that SkM-EVs are mainly accumulated in the interstitium and that they do not account for the major proportion of circulating EVs. Our findings also suggest that exercise does not promote the release of EVs from skeletal muscle.

### SkM-interstitium EVs are rich in myomiRs and promote myoblast differentiation

**Fig. 6.**
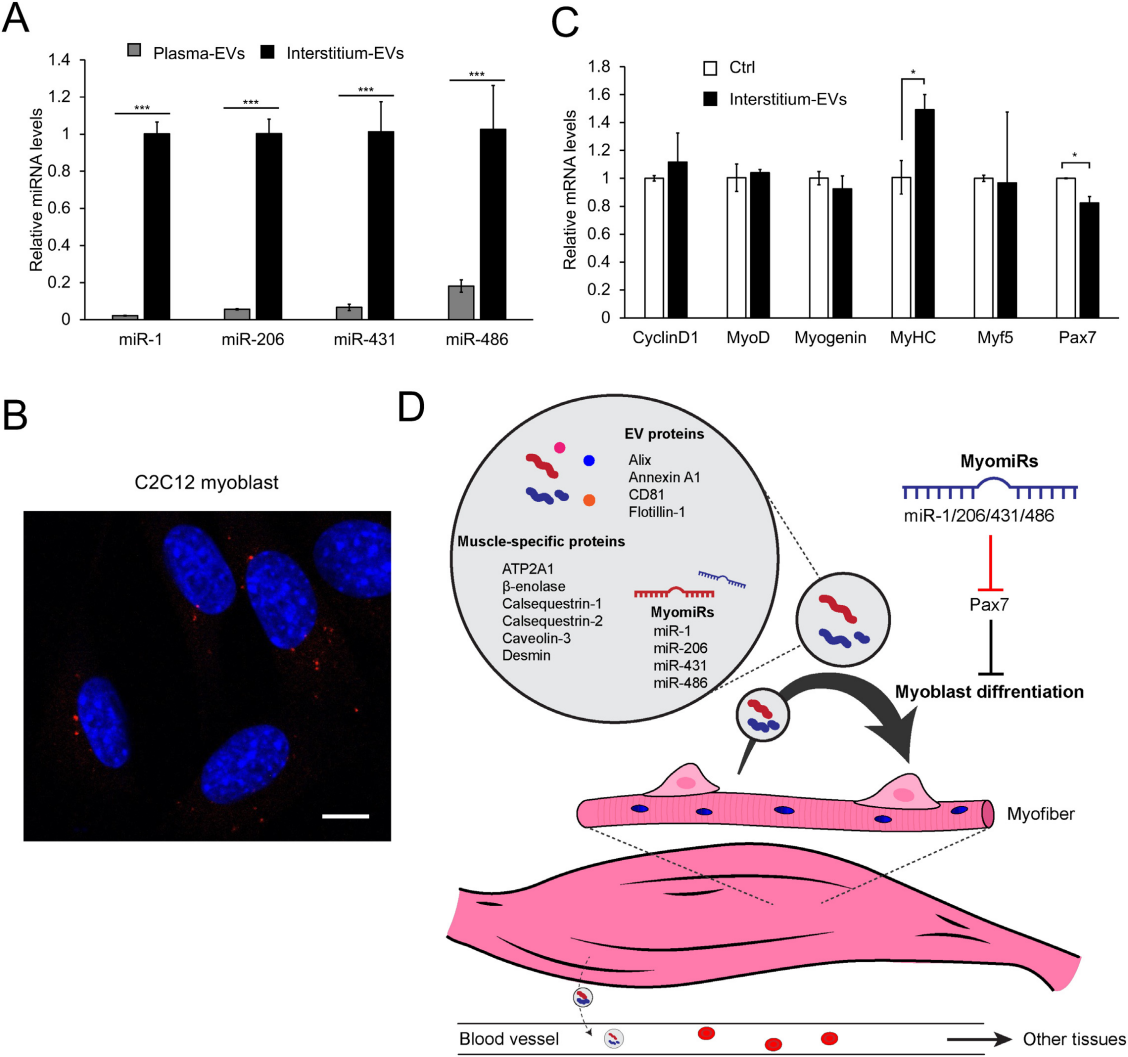
Functional analysis of SkM-interstitium EVs. (A) Exosomal miRNAs. Levels of miRs-1, -206, -431, and -486 in plasma and interstitium PS^+^ EVs were determined by qPCR as described in “Materials and Methods” section. (B) Uptake of interstitium PS^+^ EVs by myoblasts. C2C12 myoblasts were incubated with fluorescently labeled interstitium PS^+^ EVs (4 μg protein/well, red) for 6 h. After fixation, permeabilization, and nucleus staining with DAPI (blue), cell images were acquired by a confocal microscopy. Bar, 10 μm. (C) Effect of interstitium PS^+^ EVs on gene expression in myoblasts. C2C12 myoblasts were incubated with interstitium PS^+^ EVs (4 μg protein/well) in growth medium for 24 h. mRNA levels of the indicated genes were analyzed by qPCR. Results are shown as mean ± SEM (*n* = 3). Statistical analysis was performed by Student's *t*-test. * *P* < 0.05, ** *P* < 0.01, and *** *P* < 0.005. (D) A model depicting the role of SkM-EVs. See text for more detail.

EVs are characterized as the vehicle for miRNAs. Therefore, we finally investigated myomiR profiles of SkM-interstitium EVs and plasma EVs. The results show that all the four miRNAs (miRs-1, -206, -431, and -486) abundantly expressed in the muscle are markedly concentrated in the interstitium EVs (Fig. 6A). In particular, miR-1 and miR-206 in the interstitium EVs were 45- and 20-fold higher than those in plasma EVs, respectively, confirmaing the intramuscular accumulation of SkM-EV detected by our protein-based analysis. Together, these results demonstrate that SkM-interstitium EVs display unique protein and miRNA profiles that are distinct from plasma EVs. MyomiRs play important roles in skeletal muscle homeostasis, including the regulation of myogenesis by targeting the paired box transcription factor Pax7, a master regulator for myogenesis (39–41). Our results led us to hypothesize that SkM-EVs predominantly play their roles within the intramuscular microenvironment. To test this, we asked whether SkM-interstitium EVs isolated from mice modulate the expression of genes involved in myogenesis. Figure 6B shows that C2C12 myoblasts uptake the interstitium EVs, suggesting that SkM-EVs function in these cells. We next determined mRNA levels that regulate myoblast differentiation. The results show that the interstitium EVs suppress *Pax7* expression but increase *MyHC* expression, a marker for myoblast differentiation (Fig. 6C). Although the downregulation of *Pax7* by interstitium EVs was not as robust as overexpression of myomiRs in myoblasts, our results were largely consistent with previous studies showing the repression of *Pax7* mRNA expression by myomiRs (39–41). The current results thus suggest that SkM-interstitium EVs regulate myogenesis at least in part by suppressing *Pax7* expression within the muscle microenvironment (Fig. 6D).

## Discussion

Circulating EVs are derived from various sources. For identification of their origin, it is essential to determine tissue-specific EV marker molecules. Proteomic approaches have been taken to search tissue-specific EV markers using cell culture models, including skeletal muscle cells (14, 15), hepatocytes (42), and adipocytes (43). Although these studies identified potential tissue-specific EV marker proteins, their validity *in vivo* has been poorly characterized. Accordingly, much less is known about the dynamic movement of EVs in the body. In this work, we sought to identify marker proteins that help characterize EVs derived from skeletal muscle both in humans and mice. We first determined proteomic profiles of EVs released from C2C12 myoblasts, C2C12 myotubes, and hiPSC-myocytes and identified several proteins that serve as potential markers for SkM-EVs, including ATP2A1, β-enolase, and desmin. They are all predicted to reside in the luminal side of EVs. We then demonstrated that these marker proteins are relevant to identifying SkM-EVs *in vivo*. Finally, we showed that SkM-EVs accumulate within the muscle microenvironment where they regulate gene expression, rather than enter the blood circulation.

In addition to myokines secreted upon exercise, exercise-induced EVs are expected to exert health benefits (44, 45). A recent work showed that SkM-EVs from trained mice contain higher levels of miR-133b, which suppresses FoxO1 expression in the liver and improves insulin sensitivity (31). On the other hand, it was shown that skeletal muscle is not the major source of exercise-induced EVs (23). It is thus important to identify the nature of exercise-induced EVs, including their components and origins. Attempts have been made to identify markers for SkM-EVs, yet any defined markers applicable to *in vivo* analysis have not been determined at present. Several studies have reported potential markers for SkM-EVs. It was suggested that α-sarcoglycan (SGCA)-positive EVs present in the plasma are derived from skeletal muscle (46). In contrast, other studies failed to detect SGCA-positive EVs in human subjects either before or after exercise training (23). Furthermore, SGCA is not exclusively expressed in skeletal muscle but also expressed in other tissues, including the heart, smooth muscle, and lung. For the same reason, we excluded several proteins consisting of the sarcoglycan complex, including SGCD and SGCG as candidates for SkM-EV markers although these proteins were detected by our proteomic analysis. In addition, although myomiRs are found in both human and mouse plasma EVs (17), a recent report demonstrates that adipose tissue is a major source of circulating exosomal miRNAs in a context of lipodystrophy (47). On the other side, a more recent paper shows that SkM-EVs reach the blood and that skeletal muscle releases more EVs than adipose tissue *ex vivo* in a healthy context (24). These contrasting findings indicate the lack of consensus on how SkM-EVs behave after secretion and the difficulty in tracking SkM-EVs *in vivo*.

Our current analyses on proteomic profiling of human and mouse skeletal muscle cell-derived EVs combined with *in vivo* validation provide more reliable markers for SkM-EVs. Among proteins predominantly expressed in skeletal muscle, we showed that ATP2A1, β-enolase, and desmin may serve as reliable SkM-EV marker proteins. By monitoring these marker proteins, we investigated whether SkM-EVs account for a significant proportion of circulating EVs and whether exercise increases SkM-EVs *in vivo*. Unexpectedly, SkM-EVs marker proteins were hardly detected in the plasma even after exercise. Consistent with this observation, our exosomal miRNA analysis showed that myomiR levels in plasma EVs are only subtle compared to those in interstitium EVs; plasma miR-1 and -206 levels are approximately 2% and 5% of the interstitium, respectively. These results may be consistent with previous reports showing that SGCA-positive EVs constitute only 1% to 5% of total circulating EVs (46) and that most circulating exosomal miRNAs are derived from adipose tissue (47). A recent report also reached a similar conclusion; Estrada et al. (24) showed using a skeletal muscle myofiber-specific fluorescent reporter mouse model, that myofiber-derived EVs entered the blood through an unknown mechanism and accounted for 4% to 5% of plasma tetraspanin-positive EVs. Our results are also supported by evidence that exercise-induced EVs are derived from leukocytes, platelets, and endothelial cells (23) and that treadmill running does not influence muscle-specific miRNA levels in serum (48). In contrast, we found that SkM-EVs are highly accumulated in the skeletal muscle interstitium. All these results support our view that skeletal muscle is not the major source of circulating EVs regardless of physical activities and that SkM-EVs dominantly play a role within the tissue, not at systemic levels (Fig. 6D).

What is the role of SkM-EVs in the muscle microenvironment? Our data disclosed that SkM-interstitium EVs contain myomiRs (miRs-1, -206, -431, and -486) at much higher levels than plasma EVs. These myomiRs in the interstitium EVs serve as negative regulators of Pax7, leading to myoblast differentiation (39–41). We showed that SkM-interstitium EVs isolated from mice are internalized into myoblasts, suppress *Pax7* gene expression, and up-regulate *MyHC* gene expression in murine myoblasts. We thus propose that SkM-EVs support myogenesis through myomiRs-mediated suppression of Pax7, in addition to the regulation of SIRT1 (16). Although our current data showed that only subtle amounts of SkM-EVs are found in the blood, it was reported that SkM-interstitium EVs modulate hepatic gene expression when added in cultured hepatocytes or injected intravenously in mice (31). We do not exclude the possibility that small but significant amounts of SkM-EVs enter the circulation and participate in tissue communication as recently proposed (24, 31). Whether sufficient amounts of SkM-EVs are delivered to other tissues through the circulation for regulating the physiological states of recipient tissues/cells may need further investigation.

Tissues are composed of heterogeneous populations of cells, and thus the interstitium should contain EVs released from various cell types. In addition, the isolation of EVs from tissue interstitium involves gentle mincing of tissue, which may cause disruption of cells and contamination of non-EV materials. In this work, to avoid the possible contamination of membranous fragments derived from the tissue or organelles, such as the sarcolemma, and circulating lipoproteins, we isolated SkM-interstitium EVs using PS- and CD81-affinity beads after filtration with a 0.2 μm filter and compared the amounts of the marker proteins in PS^+^ and CD81^+^ EVs. These methods cannot separate myofiber-derived EVs from those secreted from other cell types, including immune cells and fibroblasts. Nevertheless, PS^+^ and CD81^+^ EVs isolated from SkM-interstitium contain myofiber-specific proteins at much higher levels than plasma, indicating that myofiber-derived EVs accumulate within the tissue. A combination of PS- or tetraspanin-capture methods with size exclusion chromatography could classify SkM-EVs more closely. Furthermore, we found that the protein compositions of PS^+^ and CD81^+^ EVs are not identical. Whether these EVs are generated by different mechanisms (e.g. exosomes vs. microvesicles) and/or exert distinct functions with specific signatures remain to be investigated. The identification of SkM-specific proteins that locate on the surface of EVs will enable us to isolate myofiber-derived EVs not only from the interstitium but also from blood. This approach can be applied to EVs released from other tissues and may also facilitate more efficient and specific isolation of interstitial EVs from tissues.

In summary, we revealed the distinct protein and miRNA profiles of SkM-EVs *in vivo*. Tracking SkM-EV markers led us to conclude that SkM-EVs do not account for the major population of circulating EVs although skeletal muscle is the largest tissue in the body. Rather, we showed that SkM-EVs highly accumulate within the skeletal muscle microenvironment where they regulate gene expression to promote myogenesis at least partially through myomiRs.

## Materials and Methods

### Materials

Fetal bovine serum (FBS) and horse serum (HS) were obtained from Gibco. FBS and HS were heat-inactivated before use. EV-free FBS and HS were prepared as described (25). Briefly, FBS and HS were spun at 2,000 *g* for 10 min followed by centrifugation at 100,000 *g* for 70 min. The supernatant was further centrifuged at 100,000 *g* for 16 h. The supernatant was filtrated with a 0.20 μm filter (Advantec) and used as EV-free FBS or HS. EV-free FBS and HS were stored at −80°C until use.

### Cell culture

C2C12 mouse myoblasts (obtained from ATCC) were maintained at low cell density in growth medium (DMEM supplemented with 10% FBS). For differentiation to myotubes, C2C12 myoblasts were seeded into a 6-well plate at a density of 1.5 × 10^5^ cells per well and grown for 2 days in a growth medium. Afterward, cells were incubated for 4 days in a differentiation medium (DMEM supplemented with 2% HS). The medium was changed every other day. For isolation of EVs, cells were incubated in 2% EV-free HS for 48 h. Human iPS cell (hiPSC) lines, 414C2^tet-Myo-D^ and 409B2^tet-MyoD^ were maintained in StemFit AK02N (Ajinomoto) as described (49). These hiPSCs were differentiated into myocytes by a published protocol (49). Briefly, on day 0, hiPSCs were seeded into a Matrigel-coated 6-well plate at a density of 3 to 4 × 10^5^ cells/well and grown overnight in StemFit medium with 10 μM Y-27632. On day 1, the medium was switched to Primate ES Cell Medium (Reprocell) containing 10 μM Y-27632. On day 2, cells were incubated in Primate ES Cell Medium containing 1 μg/mL doxycycline (Dox) to induce MyoD1 expression. On day 3, the medium was changed to αMEM containing 5% KnockOut Serum Replacement (Gibco) and Dox (1 μg/mL) and incubated for 2 to 3 days. After differentiation, hiPSC-myocytes were incubated in DMEM containing 2% EV-free HS for 48 h to isolate EVs.

### Animal studies

All protocols for animal procedures were approved by the Animal Care and Use Committee of the University of Tokyo, which are based on the Law for the Humane Treatment and Management of Animals (Law No. 105, 1 October 1973, as amended on 1 June 2020). C57BL/6 J male mice at 8 weeks old were obtained from Japan Clea. Mice were housed in a 12 h-light/12 h-dark schedule at 23 ± 2°C and 55 ± 10% humidity and fed *ad libitum* with a standard chow diet (Labo MR Stock, Nosan Corporation) and water. Mice at 9 to 10 weeks old were randomly assigned to either exercise or sedentary groups. After mice were adapted to the treadmill (5 m/min for 10 min per day) for 4 days, they were subjected to exhaustion running for up to 90 min using a ramped treadmill exercise protocol starting at 10 m/min and increasing by 2 m/min every 10 min (22) using a treadmill (MK-680C, Muromachi Kikai). Mice were defined as the exhausted state when they stopped running on a treadmill for more than 5 s despite gentle encouragement. Immediately after exercise, blood was collected by cardiac puncture under anesthesia with isoflurane. Afterward, mice were perfused through the left ventricle with PBS for 2 min at a rate of 1 mL/min to remove blood from the tissue, and skeletal muscle (tibialis anterior, gastrocnemius, soleus, and quadriceps) and other tissues were then harvested.

### Isolation of EVs from conditioned media

We used three methods to isolate EVs. Method I: EVs were isolated by ultracentrifugation according to a method previously described (25). Briefly, conditioned media (typically 12 mL from 6 wells) where cells were incubated in EV-free medium for 48 h was spun sequentially at 300 *g* for 10 min, 2,000 *g* for 10 min, and 10,000 *g* for 30 min. After each centrifugation step, the supernatant was transferred to a new centrifuge tube. The 10,000 *g*-supernatant was filtered through a 0.20 μm filter (Advantec) to obtain small EVs. Afterward, the supernatant was ultracentrifuged at 100,000 *g* for 70 min at 4°C using an MLA-55 rotor (Beckman Coulter) and an Optima MAX-TL Ultracentrifuge (Beckman Coulter). Pellet was washed once with PBS (2 mL/tube) and EVs were pelleted by ultracentrifugation at 100,000 *g* for 70 min at 4°C again. Resulting pellet was resuspended in 150 μL of PBS. Method II: The 10,000 *g*-supernatant was prepared as described above. After filtration with a 0.20 μm filter and concentration with Amicon Ultra-15 (Merck), EVs were isolated using by MagCapture Exosome Isolation Kit PS (Fujifilm-Wako) according to the manufacture instruction. This method is based on the ability of Tim4 protein to bind phosphatidylserine (PS), which localizes on the exosome surface (29). In brief, medium concentrated (1 mL) as above was mixed with 0.6 mg of streptavidin magnetic beads bound to 1 μg of biotinylated mouse  Tim4- Fc and incubated in the presence of 2 mM CaCl _2_ for 16 to 18 h with rotation at 4°C. After washing beads three times with 1 mL of washing buffer (20 mM Tris-HCl pH 7.4,150 mM NaCl, 2 mM CaCl_2_, and 0.0005% Tween20), EVs were eluted twice with 50 μL of elution buffer (20 mM Tris-HCl pH 7.4,150 mM NaCl, and 2 mM EDTA). Method III: The 10,000 *g*-supernatant prepared was filtrated and concentrated as Method II. Afterward, EVs were isolated using Exosome Isolation Kit CD81, mouse (Miltenyi Biotec) according to the manufacture's instruction. In brief, medium concentrated (1 mL) was mixed with CD81-binding magnetic beads and incubated for 1 h with rotation at room temperature. Magnetically labeled EVs were loaded onto a μ Column placed in the magnetic field of MACS Separator (Miltenyi Biotec). After washing the column, EVs were eluted with Isolation Buffer (100 μL/sample). EV protein contents were determined by Micro BCA Protein Assay Kit (Thermo Fisher). EVs were stored at −80°C until use.

### Isolation of EVs from mice

Plasma EVs were isolated by MagCapture Exosome Isolation Kit PS (Fujifilm-Wako) and Exosome Isolation Kit CD81, mouse (Miltenyi Biotec) as described above. Plasma (300 μL) from a mouse was mixed with PBS (600 μL) and spun at 10,000 *g* for 30 min. After filtration of the supernatant with a 0.20 μm filter, plasma was subjected to the isolation of EVs using MagCapture Exosome Isolation Kit PS or Exosome Isolation Kit CD81, mouse with elution volume of 100 μL per 300 μL plasma. Skeletal muscle interstitium EVs were isolated according to a method recently reported (30, 50). Skeletal muscle tissues (tibialis anterior, gastrocnemius, soleus, and quadriceps) from hind limbs of a mouse (approximately 300 mg in total) were combined and digested with collagenase (10 mg/mL, Sigma) and dispase II (10,000 PU/mL, Wako-Fujifilm) for 1 h at 37°C in HEPES buffer (100 mM HEPES and 2.5 mM CaCl_2_) immediately (within 1 h) after harvesting, to avoid excessive cell death. To avoid disruption of cells, tissues were minced gently. Afterward, one volume of PBS containing 2 mM EDTA was added to the sample, and the sample was passed through a 100 μm cell strainer (Corning). Samples were then centrifuged at 600 *g* for 5 min at 4°C, 2,000 *g* for 10 min, and 10,000 *g* for 30 min. The supernatant was filtrated with a 0.20 μm filter and concentrated using Amicon Ultra-15. EVs were then isolated by MagCapture Exosome Isolation Kit PS or Exosome Isolation Kit CD81, mouse as described above. EVs were eluted with 100 μL of elution buffer per 300 mg tissue.

### Immunoblotting and antibodies

Cells were lysed with urea buffer (8 M Urea, 50 mM Na-phosphate pH 8.0, 10 mM Tris-HCl pH 8.0, and 100 mM NaCl) containing protease inhibitor cocktail (Nacalai Tesque) as described (51). Tissue homogenates were prepared in radioimmunoprecipitation assay buffer (50 mM Tris-HCl pH 7.4, 150 mM NaCl, 1 mM EDTA, 1% Nonidet P-40, and 0.25% sodium deoxycholate) supplemented with a protease inhibitor mixture (Nacalai Tesque) and phosphatase inhibitor mixture (Sigma). Protein concentration was determined by BCA Protein Assay (Thermo Fisher). Cell lysate, tissue homogenate, and EVs were mixed with Laemmli buffer and heated at 95°C for 3 min. Aliquots were subjected to SDS-PAGE and immunoblot analysis according to a standard protocol. The expression of a protein was analyzed by Image J software or Evolution-Capt software (Vilber Lourmat). Antibodies used were obtained from commercial sources as follows: anti-caveolin 3 (sc-5310), anti-calsequestrin 1 (sc-137080), anti-calsequestrin 2 (sc-390999), anti-CD81 (sc-166029), anti-β enolase (sc-100811), anti-HSP90 (sc-13119), anti-Calnexin (sc-46669), anti-TSG101 (sc-7964) antibodies from Santa Cruz Biotechnology; anti-flotillin 1 antibody (ab41927) from Abcam; anti-CD81 (#10037), anti-Alix (#92880), anti-desmin (#5332), anti-Tom20 (#42406), anti-ATP2A1 (#12293), anti-GAPDH (#5174), HRP-linked anti-mouse IgG (#7076), and HRP-linked anti-rabbit IgG (#7074) antibodies from Cell Signaling Technology; anti-apolipoprotein A-I rabbit mAb (14227–1-AP) from Proteintech; anti-apolipoprotein E mouse mAb (NB110-60531) from Novus Biologicals; anti-GM130 (610823) from BD Bioscience; anti-myosin heavy chain, clone # MF20 (MAB4470) from R&D Systems; Mouse TrueBlot: Anti-Mouse Ig HRP (18–8817–31) from Rockland Immunochemicals.

### Proteomic analysis of EVs

EVs were solubilized in 50 mM Tris-HCl pH 9.0 containing 5% sodium deoxycholate, reduced with 10 mM dithiothreitol for 60 min at 37°C, and alkylated with 55 mM iodoacetamide for 30 min in the dark at 25°C. The reduced and alkylated samples were diluted 10-fold with 50 mM Tris-HCl pH 9.0 and digested with trypsin at 37°C for 16 h (trypsin-to-protein ratio of 1:20 (w/w)). An equal volume of ethyl acetate was added to each sample solution and the mixtures were acidified with the final concentration of 0.5% trifluoroacetic acid. The mixtures were shaken for 1 min and centrifuged at 15,700 g for 2 min. The aqueous phase was collected and desalted with C18-StageTips. LC-MS/MS analysis was performed using an UltiMate 3000 Nano LC system (Thermo Fisher Scientific) coupled to Orbitrap Fusion Lumos hybrid quadrupole-Orbitrap mass spectrometer (Thermo Fisher Scientific) with a nano-electrospray ionization source. The sample was injected by an autosampler and enriched on a C18 reverse-phase trap column (100 μm × 5 mm length, Thermo Fisher Scientific) at a flow rate of 4 μL/min. The sample was subsequently separated by a C18 reverse-phase column (75 μm × 150 mm length, Nikkyo Technos) at a flow rate of 300 nL/min with a linear gradient from 2% to 35% mobile phase B (95% acetonitrile and 0.1% formic acid). The peptides were ionized using nano-electrospray ionization in positive ion mode. The raw data were analyzed by Mascot Distiller v2.3 (Matrix Science), and peak lists were created based on the recorded fragmentation spectra. Peptides and proteins were identified by Mascot v2.3 (Matrix Science) using UniProt database with a precursor mass tolerance of 10 ppm, a fragment ion mass tolerance of 0.01 Da and strict trypsin specificity allowing for up to 1 missed cleavage. The carbamidomethylation of cysteine and the oxidation of methionine were allowed as variable modification.

### Electron microscopy

Specimens for transmission electron microscopy (TEM) were prepared at room temperature. An aliquot of EV sample was pipetted onto a copper grid with carbon support film and incubated for 10 min. After the excess liquid was removed, a grid was briefly placed on 10 μL 2% uranyl acetate (w/v, Merck). Images were acquired under a JEM-1010 electron microscope (JEOL) operated at 100 kV with a Keen view CCD camera (Olympus Soft Imaging Solution). The size of EVs was measured using Image J software.

For scanning electron microscopy (SEM) analysis, skeletal muscle tissue (approximately 3 × 3 mm in size) was fixed with 10% neutral buffered formalin for 1 h and with 0.2% glutaraldehyde and 2% paraformaldehyde in PBS for 1 h. After post-fixation with 1% osmium tetroxide in PBS, samples were dehydrated in ethanol series (70%, 90%, 95%, 99.5%, and 100%) for 10 min each, treated with tert-butyl alcohol for 10  min twice and freeze-dried. The dried specimen was applied onto a carbon double side-tape with silver paste and sputter coated with platinum palladium. Images were acquired under a Hitachi S-4800 scanning electron microscope with a secondary electron in-lens detector.

### EV labeling and confocal microscopy

EVs were labeled using ExoSparkler Exosome Protein Labeling Kit-Red (Dojindo Laboratories) according to the manufacturer's instruction. C2C12 myoblasts seeded in a 35-mm film bottom dish (Matsunami) were incubated without or with the labeled EVs (4 μg protein per 2 mL). Cells were fixed with 4% paraformaldehyde (Fujifilm-Wako) for 10 min and then permeabilized with 0.1% Triton X-100 in PBS for 5 min at room temperature. After nuclei were stained with DAPI, specimens were mounted with ProLong Gold Antifade Reagent (Thermo Fisher). Cell images were acquired by an LSM800 confocal laser microscope (Carl Zeiss) equipped with a Plan-Apochromat 63×/1.4 objective. Images were processed with a Zen software (Carl Zeiss).

### Immunostaining

C2C12 myotubes and hiPSC-myocytes (both differentiated in a 35-mm film bottom dish) were fixed and permeabilized as above. After blocking with 5% FBS in PBS for 1 h, specimens were incubated with anti-MHC antibody (1:100 dilution, R&D Systems) for 1 h followed by incaubation with Alexa Fluor 568-conjugated anti-mouse IgG (1:800 dilution, Thermo Fisher) for 45 min. Nuclei were stained with DAPI, and specimens were mounted as above. Cell images were obtained by an LSM800 confocal laser microscope (Carl Zeiss).

### mRNA expression analysis

Total RNA was isolated using ISOGEN (NIPPON GENE), according to the manufacturer's instructions. The high-capacity cDNA reverse transcription kit (Applied Biosystems) was used to synthesize cDNA from total RNA. Quantitative real-time PCR (qPCR) analyses were performed using an Applied Biosystems StepOnePlus. mRNA levels were normalized to 18S ribosomal RNA levels. The primers used for qPCR analysis are described in *SI Appendix*, Table S6.

### miRNA analysis

Plasma and SkM-interstitium EVs were isolated from two mice and pooled for miRNA analyses. Total RNA was extracted from EVs (20 μg protein) using miRNeasy Mini Kit (Qiagen). RNA was then reverse-transcribed using TaqMan MicroRNA Reverse Transcription Kit (Applied Biosystems) according to the manufacturer's protocol. qPCR was then performed using TaqMan MicroRNA Assay (miR-1, Assay ID: 002222; miR-206, Assay ID: 000510; miR-431, Assay ID: 001979; miR-486, Assay ID: 002093; miR-16, Assay ID: 000391; miR-21, and Assay ID: 000397) (Applied Biosystems). Exosomal miRNA levels were normalized by the mean value of miR-16 and miR-21 as described (31).

### Statistical analysis

Results are expressed as mean ± SEM from at least three independent biological replicates. Statistical analyses were performed using the two-tailed, unpaired Student's *t*-test. *P* values less than 0.05 were considered statistically significant.

## Funding

This work was supported by KAKENHI grants 19H02908, 22H02281 (to Y.Y.), and 20H00408 (to R.S.) from the Japan Society for the Promotion of Science, and AMED-CREST grants 20gm091008h and 21gm091008h (to Y.Y. and R.S.) from the Japan Agency for Medical Research and Development. S.W. was supported by the Japan Society for the Promotion of Science Research Fellowship for Young Scientists (20J22415).

## Preprint Server

This manuscript has been deposited on bioRxiv (doi: https://doi.org/10.1101/2021.11.30.470551).

## Supplementary Material

pgac173_Supplemental_Files

## Data Availability

All data are included in the article and Supplementary data.

## References

[1] Pedersen BK , Febbraio MA. 2012. Muscles, exercise and obesity: skeletal muscle as a secretory organ. Nat Rev Endocrinol. 8:457–465. 22473333 10.1038/nrendo.2012.49

[2] Hoffmann C , Weigert C. 2017. Skeletal muscle as an endocrine organ: the role of myokines in exercise adaptations. Cold Spring Harb Perspect Med. 7:a029793. 28389517 10.1101/cshperspect.a029793 PMC5666622

[3] Ruegsegger GN , Booth FW. 2018. Health benefits of exercise. Cold Spring Harb Perspect Med. 8:a029694. 28507196 10.1101/cshperspect.a029694 PMC6027933

[4] van Niel G , D'Angelo G, Raposo G. 2018. Shedding light on the cell biology of extracellular vesicles. Nat Rev Mol Cell Biol. 19:213–228. 29339798 10.1038/nrm.2017.125

[5] Record M , Silvente-Poirot S, Poirot M, Wakelam MJO. 2018. Extracellular vesicles: lipids as key components of their biogenesis and functions. J Lipid Res. 59:1316–1324. 29764923 10.1194/jlr.E086173 PMC6071772

[6] Mathieu M , Martin-Jaular L, Lavieu G, Théry C. 2019. Specificities of secretion and uptake of exosomes and other extracellular vesicles for cell-to-cell communication. Nat Cell Biol. 21:9–17. 30602770 10.1038/s41556-018-0250-9

[7] Pegtel DM , Gould SJ. 2019. Exosomes. Annu Rev Biochem. 88:487–514. 31220978 10.1146/annurev-biochem-013118-111902

[8] O'Brien K , Breyne K, Ughetto S, Laurent LC, Breakefield XO. 2020. RNA delivery by extracellular vesicles in mammalian cells and its applications. Nat Rev Mol Cell Biol. 21:585–606. 32457507 10.1038/s41580-020-0251-y PMC7249041

[9] Pitt JM , Kroemer G, Zitvogel L. 2016. Extracellular vesicles: masters of intercellular communication and potential clinical interventions. J Clin Invest. 126:1139–1143. 27035805 10.1172/JCI87316 PMC4811136

[10] Kalluri R , LeBleu VS. 2020. The biology, function, and biomedical applications of exosomes. Science. 367:640. 10.1126/science.aau6977 PMC7717626 32029601

[11] Mori MA , Ludwig RG, Garcia-Martin R, Brandão BB, Kahn CR. 2019. Extracellular miRNAs: from biomarkers to mediators of physiology and disease. Cell Metab. 30:656–673. 31447320 10.1016/j.cmet.2019.07.011 PMC6774861

[12] Rome S , Forterre A, Mizgier ML, Bouzakri K. 2019. Skeletal muscle-released extracellular vesicles: state of the art. Front Physiol. 10:929. 31447684 10.3389/fphys.2019.00929 PMC6695556

[13] Guescini M et al. 2010. C2C12 myoblasts release micro-vesicles containing mtDNA and proteins involved in signal transduction. Exp Cell Res. 316:1977–1984. 20399774 10.1016/j.yexcr.2010.04.006

[14] Le Bihan MC et al. 2012. In-depth analysis of the secretome identifies three major independent secretory pathways in differentiating human myoblasts. J Proteomics. 77:344–356. 23000592 10.1016/j.jprot.2012.09.008

[15] Forterre A et al. 2014. Proteomic analysis of C2C12 myoblast and myotube exosome-like vesicles: a new paradigm for myoblast-myotube cross talk?. PLoS One. 9:e84153. 24392111 10.1371/journal.pone.0084153 PMC3879278

[16] Forterre A et al. 2014. Myotube-derived exosomal miRNAs downregulate Sirtuin1 in myoblasts during muscle cell differentiation. Cell Cycle. 13:78–89. 24196440 10.4161/cc.26808 PMC3925739

[17] Matsuzaka Y et al. 2016. Characterization and functional analysis of extracellular vesicles and muscle-abundant miRNAs (miR-1, miR-133a, and miR-206) in C2C12 myocytes and mdx mice. PLoS One. 11:e0167811. 27977725 10.1371/journal.pone.0167811 PMC5158003

[18] Horak M , Novak J, Bienertova-Vasku J. 2016. Muscle-specific microRNAs in skeletal muscle development. Dev Biol. 410:1–13. 26708096 10.1016/j.ydbio.2015.12.013

[19] Giagnorio E , Malacarne C, Mantegazza R, Bonanno S, Marcuzzo S. 2021. MyomiRs and their multifaceted regulatory roles in muscle homeostasis and amyotrophic lateral sclerosis. J Cell Sci. 134.jcs258349 34137441 10.1242/jcs.258349

[20] Garcia-Martin R , Brandao BB, Thomou T, Altindis E, Kahn CR. 2022. Tissue differences in the exosomal/small extracellular vesicle proteome and their potential as indicators of altered tissue metabolism. Cell Rep. 38:110277. 35045290 10.1016/j.celrep.2021.110277 PMC8867597

[21] Frühbeis C , Helmig S, Tug S, Simon P, Krämer-Albers EM. 2015. Physical exercise induces rapid release of small extracellular vesicles into the circulation. J Extracell Vesicles. 4:28239. 26142461 10.3402/jev.v4.28239 PMC4491306

[22] Whitham M et al. 2018. Extracellular vesicles provide a means for tissue crosstalk during exercise. Cell Metab. 27:237–251. 29320704 10.1016/j.cmet.2017.12.001

[23] Brahmer A et al. 2019. Platelets, endothelial cells and leukocytes contribute to the exercise-triggered release of extracellular vesicles into the circulation. J Extracell Vesicles. 8:1615820. 31191831 10.1080/20013078.2019.1615820 PMC6542154

[24] Estrada AL et al. 2022. Extracellular vesicle secretion is tissue-dependent ex vivo and skeletal muscle myofiber extracellular vesicles reach the circulation in vivo. Am J Physiol Cell Physiol. 322:C246–C259. 34910603 10.1152/ajpcell.00580.2020 PMC8816621

[25] Thery C , Amigorena S, Raposo G, Clayton A. 2006. Isolation and characterization of exosomes from cell culture supernatants and biological fluids. Curr Protoc Cell Biol. Chapter 3:Unit 3 22. 10.1002/0471143030.cb0322s30 18228490

[26] Pathan M et al. 2019. Vesiclepedia 2019: a compendium of RNA, proteins, lipids and metabolites in extracellular vesicles. Nucleic Acids Res. 47:D516–D519. 30395310 10.1093/nar/gky1029 PMC6323905

[27] Huang dW , Sherman BT, Lempicki RA. 2009. Systematic and integrative analysis of large gene lists using DAVID bioinformatics resources. Nat Protoc. 4:44–57. 19131956 10.1038/nprot.2008.211

[28] Huang dW , Sherman BT, Lempicki RA. 2009. Bioinformatics enrichment tools: paths toward the comprehensive functional analysis of large gene lists. Nucleic Acids Res. 37:1–13. 19033363 10.1093/nar/gkn923 PMC2615629

[29] Nakai W et al. 2016. A novel affinity-based method for the isolation of highly purified extracellular vesicles. Sci Rep. 6:33935. 27659060 10.1038/srep33935 PMC5034288

[30] Crewe C et al. 2018. An endothelial-to-adipocyte extracellular vesicle axis governed by metabolic state. Cell. 175:695–708. 30293865 10.1016/j.cell.2018.09.005 PMC6195477

[31] Castano C , Mirasierra M, Vallejo M, Novials A, Parrizas M. 2020. Delivery of muscle-derived exosomal miRNAs induced by HIIT improves insulin sensitivity through down-regulation of hepatic FoxO1 in mice. Proc Natl Acad Sci USA. 117:30335–30343. 33199621 10.1073/pnas.2016112117 PMC7720135

[32] Théry C et al. 2018. Minimal information for studies of extracellular vesicles 2018 (MISEV2018): a position statement of the International Society for Extracellular Vesicles and update of the MISEV2014 guidelines. J Extracell Vesicles. 7:1535750. 30637094 10.1080/20013078.2018.1535750 PMC6322352

[33] Takov K , Yellon DM, Davidson SM. 2019. Comparison of small extracellular vesicles isolated from plasma by ultracentrifugation or size-exclusion chromatography: yield, purity and functional potential. J Extracell Vesicles. 8:1560809. 30651940 10.1080/20013078.2018.1560809 PMC6327926

[34] Brennan K et al. 2020. A comparison of methods for the isolation and separation of extracellular vesicles from protein and lipid particles in human serum. Sci Rep. 10:1039. 31974468 10.1038/s41598-020-57497-7 PMC6978318

[35] Dong L et al. 2020. Comprehensive evaluation of methods for small extracellular vesicles separation from human plasma, urine and cell culture medium. J Extracell Vesicles. 10:e12044. 33489012 10.1002/jev2.12044 PMC7810129

[36] Oliveira GP et al. 2018. Effects of acute aerobic exercise on rats serum extracellular vesicles diameter, concentration and small RNAs content. Front Physiol. 9:532. 29881354 10.3389/fphys.2018.00532 PMC5976735

[37] Lovett JAC , Durcan PJ, Myburgh KH. 2018. Investigation of circulating extracellular vesicle microRNA following two consecutive bouts of muscle-damaging exercise. Front Physiol. 9:1149. 30177888 10.3389/fphys.2018.01149 PMC6109634

[38] Rigamonti AE et al. 2020. Effects of an acute bout of exercise on circulating extracellular vesicles: tissue-, sex-, and BMI-related differences. Int J Obes (Lond). 44:1108–1118. 31578459 10.1038/s41366-019-0460-7

[39] Chen JF et al. 2010. microRNA-1 and microRNA-206 regulate skeletal muscle satellite cell proliferation and differentiation by repressing Pax7. J Cell Biol. 190:867–879. 20819939 10.1083/jcb.200911036 PMC2935565

[40] Dey BK , Gagan J, Dutta A. 2011. miR-206 and -486 induce myoblast differentiation by downregulating Pax7. Mol Cell Biol. 31:203–214. 21041476 10.1128/MCB.01009-10 PMC3019853

[41] Wu R et al. 2015. MicroRNA-431 accelerates muscle regeneration and ameliorates muscular dystrophy by targeting Pax7 in mice. Nat Commun. 6:7713. 26151913 10.1038/ncomms8713

[42] Conde-Vancells J et al. 2008. Characterization and comprehensive proteome profiling of exosomes secreted by hepatocytes. J Proteome Res. 7:5157–5166. 19367702 10.1021/pr8004887 PMC2696236

[43] Durcin M et al. 2017. Characterisation of adipocyte-derived extracellular vesicle subtypes identifies distinct protein and lipid signatures for large and small extracellular vesicles. J Extracell Vesicles. 6:1305677. 28473884 10.1080/20013078.2017.1305677 PMC5405565

[44] Safdar A , Saleem A, Tarnopolsky MA. 2016. The potential of endurance exercise-derived exosomes to treat metabolic diseases. Nat Rev Endocrinol. 12:504–517. 27230949 10.1038/nrendo.2016.76

[45] Vechetti IJ , Valentino T, Mobley CB, McCarthy JJ. 2021. The role of extracellular vesicles in skeletal muscle and systematic adaptation to exercise. J Physiol. 599:845–861. 31944292 10.1113/JP278929 PMC7363579

[46] Guescini M et al. 2015. Muscle releases alpha-sarcoglycan positive extracellular vesicles carrying miRNAs in the bloodstream. PLoS One. 10:e0125094. 25955720 10.1371/journal.pone.0125094 PMC4425492

[47] Thomou T et al. 2017. Adipose-derived circulating miRNAs regulate gene expression in other tissues. Nature. 542:450–455. 28199304 10.1038/nature21365 PMC5330251

[48] Mizuno H et al. 2011. Identification of muscle-specific microRNAs in serum of muscular dystrophy animal models: promising novel blood-based markers for muscular dystrophy. PLoS One. 6:e18388. 21479190 10.1371/journal.pone.0018388 PMC3068182

[49] Uchimura T , Otomo J, Sato M, Sakurai H. 2017. A human iPS cell myogenic differentiation system permitting high-throughput drug screening. Stem Cell Res. 25:98–106. 29125995 10.1016/j.scr.2017.10.023

[50] Crescitelli R , Lässer C, Lötvall J. 2021. Isolation and characterization of extracellular vesicle subpopulations from tissues. Nat Protoc. 16:1548–1580. 33495626 10.1038/s41596-020-00466-1

[51] Yamauchi Y , Furukawa K, Hamamura K. 2011. Positive feedback loop between PI3K-Akt-mTORC1 signaling and the lipogenic pathway boosts Akt signaling: induction of the lipogenic pathway by a melanoma antigen. Cancer Res. 71:4989–4997. 21632551 10.1158/0008-5472.CAN-10-4108

