# Correction to: Skeletal muscle releases extracellular vesicles with distinct protein and microRNA signatures that function in the muscle microenvironment

**DOI:** 10.1093/pnasnexus/pgad071

**Published:** 2023-03-14

This is a correction to: Sho Watanabe, Yuri Sudo, Takumi Makino, Satoshi Kimura, Kenji Tomita, Makoto Noguchi, Hidetoshi Sakurai, Makoto Shimizu, Yu Takahashi, Ryuichiro Sato, Yoshio Yamauchi, Skeletal muscle releases extracellular vesicles with distinct protein and microRNA signatures that function in the muscle microenvironment, *PNAS Nexus*, Volume 1, Issue 4, September 2022, pgac173, https://doi.org/10.1093/pnasnexus/pgac173

In the originally published version of this manuscript, the image for Calnexin was duplicated in Figure 4E's right panel (CD81-isolation) from the left panel.

The figure has now been corrected in the article.

